# Contrasting Manual and Automated Assessment of Thermal Stress Responses and Larval Body Size in Black Soldier Flies and Houseflies

**DOI:** 10.3390/insects12050380

**Published:** 2021-04-22

**Authors:** Stine Frey Laursen, Laura Skrubbeltrang Hansen, Simon Bahrndorff, Hanne Marie Nielsen, Natasja Krog Noer, David Renault, Goutam Sahana, Jesper Givskov Sørensen, Torsten Nygaard Kristensen

**Affiliations:** 1 Section of Biology and Environmental Science, Department of Chemistry and Bioscience, Aalborg University, Fredrik Bajers Vej 7H, 9220 Aalborg, Denmark; sba@bio.aau.dk (S.B.); nkn@bio.aau.dk (N.K.N.); tnk@bio.aau.dk (T.N.K.); 2 Center for Quantitative Genetics and Genomics, Faculty of Technical Sciences, Aarhus University, Blichers Allé 20, 8830 Tjele, Denmark; lsh@qgg.au.dk (L.S.H.); hannem.nielsen@qgg.au.dk (H.M.N.); goutam.sahana@qgg.au.dk (G.S.); 3 University of Rennes, CNRS, ECOBIO (Ecosystémes, Biodiversité, Evolution)-UMR, 6553 Rennes, France; david.renault@univ-rennes1.fr; 4 Institut Universitaire de France, 1 Rue Descartes, CEDEX 05, 75231 Paris, France; 5 Section for Genetics, Ecology and Evolution, Department of Biology, Aarhus University, Ny Munkegade 116, 8000 Aarhus C, Denmark; jesper.soerensen@bio.au.dk; 6 Department of Agroecology, Aarhus University, Blichers Allé 20, 8830 Tjele, Denmark

**Keywords:** heat and cold tolerance, acclimation, automated phenotyping, *Musca domestica*, *Hermetia illucens*

## Abstract

**Simple Summary:**

Studying insect physiology frequently involves assessment of certain characteristics including body size and temperature tolerance. Information on organismal traits such as body size and temperature tolerance is typically obtained by weighing or measuring individuals or by visual observation of time points or temperatures where insects lose the ability to move when exposed to stressful temperatures. Such manual methods can be time consuming, tedious and prone to human error. Therefore, accurate automated methods, with the potential to increase the number of analysed individuals, are needed. In this study, we used image analysis software to measure thermal tolerances and larval body sizes in houseflies and black soldier flies and compared the results with those obtained manually. We found a strong correlation between larval body sizes measured using the two methods and were able to decrease the time spent on the measurements markedly. We did not find the same values for temperature tolerance using the two methods, but we often found the same relationship between groups that we compared. In addition, the automated method allowed us to track the movements of the flies over long periods of time, thus increasing the information output from the experiments. We conclude that implementation of automated methods will bring new opportunities within insect research.

**Abstract:**

Within ecophysiological and genetic studies on insects, morphological and physiological traits are commonly assessed and phenotypes are typically obtained from manual measurements on numerous individuals. Manual observations are, however, time consuming, can introduce observer bias and are prone to human error. Here, we contrast results obtained from manual assessment of larval size and thermal tolerance traits in black soldier flies (*Hermetia illucens*) and houseflies (*Musca domestica*) that have been acclimated under three different temperature regimes with those obtained automatically using an image analysis software (Noldus EthoVision XT). We found that (i) larval size estimates of both species, obtained by manual weighing or by using the software, were highly correlated, (ii) measures of heat and cold tolerance using manual and automated approaches provided qualitatively similar results, and (iii) by using the software we obtained quantifiable information on stress responses and acclimation effects of potentially higher ecological relevance than the endpoint traits that are typically assessed when manual assessments are used. Based on these findings, we argue that automated assessment of insect stress responses and largescale phenotyping of morphological traits such as size will provide new opportunities within many disciplines where accurate and largescale phenotyping of insects is required.

## 1. Introduction

High-throughput technologies have rapidly become an essential part of experimental biology because they enable researchers to perform large-scale, time-efficient and streamlined experiments and analyses [1,2]. However, technological advances at the organismal phenotyping level in insects are lagging behind, and we propose that phenotyping of fitness components is progressively becoming a bottleneck in, e.g., ecophysiological and evolutionary studies on insects. In this context, unbiased automated, or semi-automated, methodologies aiming at phenotyping arthropods have been developed [3,4,5,6,7,8,9,10]. Despite these recent technological advances, the area is still in its infancy, and there is a need to automate the phenotyping process to reduce bias and increase throughput across biological disciplines.

In insects, body size is a frequently employed morphometric trait, partly due to size being correlated with several fitness components, including longevity, fecundity, metabolic rates, ability to migrate, competitive and predatory ability and abiotic stress tolerance (e.g., [11]). Information on insect body size can be easily obtained by weighing or measuring individuals, and body size is often a highly reproducible metric. With climate change, much focus has been on understanding species responses and thus quantifying environmental stress tolerance, such as resistance and response to desiccation and thermal stress [12]. Measures of thermal tolerance are frequently derived from the evaluation of lethal and non-lethal endpoint traits such as thermal limits, where death, coma or recovery following temperature stress occur. Unfortunately, obtaining measurements of size and stress tolerance traits can be labour-intensive and time-consuming, especially when the number of individuals to be handled is high. Additionally, assessing thermal tolerance endpoints in insects can be prone to observer bias [13]. Finally, there are constraints on which traits can be studied with manual observation, and while thermal limits can partially be used to predict species distributions, such measures cover only part of the ecologically important variation in insect thermal stress responses [14,15,16,17]. As climatic extremes increase in magnitude and frequency, so does the intensity of environmental stress for many species [18,19]. To predict species vulnerability and future distributions under such scenarios, the collection of reproducible measurements of fitness components is crucial. This emphasises the need for developing streamlined and holistic methods to study such traits in insects.

In our study, we investigate the potential of performing large-scale automated phenotyping of larval size and thermal stress response traits on black soldier flies (*Hermetia illucens* L.) and houseflies (*Musca domestica* L.). The housefly has a long history as a laboratory model species. In addition, both species are considered suitable species for commercial insect production [20], where accurate phenotyping of numerous individuals is required to optimise production traits. Therefore, these two fly species are obvious test candidates for high-throughput phenotyping. To investigate the applicability of automated insect phenotyping of morphological traits, we compare automated and manual phenotyping methods for larval size estimation. Furthermore, we evaluate thermal stress tolerances by exploring the critical thermal maximum (CTmax), heat knockdown time (HKDT) and chill coma recovery time (CCRT) for each species, using temperature acclimation to induce variation in these traits. We apply image analysis software (EthoVision XT) to analyse video recordings of thermal tolerance assays and compare results with manual observations of those same recordings. We hypothesize that (i) there is a strong, positive correlation between larval body size, measured manually by weighing, and surface area measured automatically, (ii) similar estimates of thermal tolerance can be obtained using manual and automated observations of first or last movement and first or last time crossing the middle of the arena, and (iii) the automated phenotyping method can provide additional insights into thermal stress responses that cannot be obtained from manual observations.

## 2. Materials and Methods

### 2.1. Fly Stock Populations

Black soldier fly larvae, used for assessment of larval body size, were obtained from Enorm Biofactory A/S (8762 Flemming, Denmark), and size was estimated immediately after receipt. Black soldier flies used for thermal stress assessment were received as larvae from Entomass ApS (9480 Løkken, Denmark) and kept in the laboratory at 27 °C with a photoperiod of 12:12 h (light:dark) for one generation prior to being used for the experiments. From the adult flies, eggs were collected over 4 days and transferred to a larval rearing medium composed of chicken feed (product code: 7005397, Danish Agro Shoppen A/S) mixed with tap water. When most larvae had reached the pupal stage, they were transferred to a cage (L: 60 cm, H: 60 cm, W: 60 cm) until emergence of the first adults. Pupae transfer was conducted at 24 h intervals, using different cages, to control the age of flies used in the experiments. Flies that emerged outside the 24 h timespan were included in the experiments only when an insufficient number of male flies had emerged during the 24 h. Adult flies had access to tap water via a dental cotton roll fixed in the lid of a water-filled plastic container.

Houseflies were hand-collected from a pig farm in Aalborg East, Denmark, in 2018 and were since held under standard laboratory conditions at 23 °C with a photoperiod of 12:12 h (light:dark). Adult flies had access to sugar, icing sugar, powdered milk and tap water. Eggs were collected over 4 h and transferred to a medium composed of wheat bran, alfalfa meal, dry yeast, malt and tap water. After hatching, the rearing medium containing larvae was regularly stirred until pupation of the larvae (for details on rearing see Kjærsgaard et al. [16]). Eight days after oviposition, larvae used for body size estimation were removed from the rearing medium. For the study of thermal stress responses, pupae were transferred from the rearing medium to Petri dishes which were transferred to an empty cage (L: 30 cm, H: 30 cm, W: 30 cm) every 12 h to keep track of the age of the emerging flies.

### 2.2. Larval Body Size

Body sizes of black soldier fly and housefly larvae were measured with a manual and an automated phenotyping method. For the manual assessment, 150 larvae of each species were weighed individually three times (technical replicates) on a precision balance (QUINTIX35-1S, Sartorius, Goettingen, Germany) and mean body mass of each individual was computed from these technical replicates, yielding one value per individual. Prior to the assessment, excess rearing medium was removed from the larvae with a paper towel. Immediately thereafter, the same 150 larvae were distributed in five flat-bottomed 30-well plates (well dimensions: H: 17 mm, Ø: 30 mm) with one individual in each well for automated size assessment. Each plate was positioned on a sheet of glass with a light box (LED Acrylic Panel A4, HUION, Mavčiče, Slovenia) placed approximately 50 cm above a video camera (25 frames per second, resolution: 1280 × 1024) (acA 1300–60 gm GigE, Basler, Ahrensburg, Germany; lens: C-mount 4–8 mm, Computar, Tokyo, Japan). The camera was connected to a computer (Figure S1). Before the transfer of larvae to the well plates, a reference image of an empty plate was acquired, and a calibration scale was defined. Mean surface area for each larva was acquired using live imaging over 60 s per plate (base version of EthoVision XT 15.0.1418 with a JavaScript enabling size estimation, Noldus, Wageningen, The Netherlands). This acquisition was repeated three times (three technical replicates) and the three mean size estimates were averaged for each larva. An acquisition time of 60 s was chosen based on preliminary pilot experiments; when evaluating results obtained using five different acquisition times, the 60 s acquisition time did not yield size estimates different from those obtained with longer or shorter acquisition times (Figure S2).

### 2.3. Thermal Stress Response Assessment

#### 2.3.1. Thermal Acclimation Treatments

For both species, 60 newly emerged adult male flies were collected (Table S1) and isolated in plastic vials (H: 95 mm, Ø: 25 mm) sealed with foam stoppers. A 10% sucrose solution was available to all flies via 1.5 mL Eppendorf tubes sealed with cotton wool stoppers and fixed in the foam stopper of each vial. The vials were placed in climatic chambers (KBWF720 and KB400, Binder, Tuttlingen, Germany) with a photoperiod of 12:12 h (light:dark) at three different species-specific acclimation treatments (low, intermediate and high temperatures) for 48 h. Acclimation temperatures were 15, 27 and 36 °C for the black soldier fly and 10, 23 and 32 °C for the housefly and the relative humidity varied from 44% to 83% between treatments. The treatment temperatures were selected based on pilot experiments from our laboratory where survival under different acclimation temperatures was tested (Table S3). They were further based on studies on other insect species revealing that adult acclimation to temperatures approximately 8–12 °C lower or higher than standard rearing temperatures induces increased thermal tolerance [21,22].

#### 2.3.2. Thermal Stress Response Assays

Prior to all assays, flies were individually transferred to 10 mL screw cap glass vials (H: 46 mm, Ø: 22 mm) via a funnel. The vials were mounted to a rack made from two semi-transparent white acrylic sheets (L: 600 mm, H: 300 mm, W: 4 mm, light transmission: 75%) with an LED light source (Flexstrip IP68, 4000K, LightPartner, Herning, Denmark) placed between the sheets (Figure S3). Ten flies from each of the three acclimation treatments were randomly distributed on each sheet, yielding a total of 60 flies for each assay (*n* = 20 flies for each treatment group). The rack was submerged in a temperature-controlled water bath (Proline 1845, LAUDA, Lauda-Königshofen, Germany) with assay- and species-specific temperatures. Chill coma recovery time (CCRT) was assessed using a static low-temperature assay, while heat knockdown time (HKDT) and critical thermal maximum (CTmax) were investigated using static high-temperature and dynamic (ramping) assays, respectively (for general information on these different assays see Terblanche et al. [23] and Overgaard et al. [24]). In the static low-temperature assays, all flies were initially forced into chill coma by submerging the rack in a water bath at 0 °C for 2 h. Subsequently, the rack with flies was transferred to another water bath with temperatures of 27 °C for black soldier flies and 23 °C for houseflies in which recovery time was monitored. In the dynamic assays, flies were subjected to a gradually increasing temperature using a ramping rate of 0.2 °C min^−1^ starting from 27 °C for black soldier flies and 23 °C for houseflies. A relatively high ramping rate was chosen to avoid confounding effects of other types of stress caused by prolonged isolation in vials and to reduce the potential for temperature hardening during ramping [25,26]. In the static high-temperature assays, black soldier flies and houseflies were exposed to a static high temperature of 46.5 and 45 °C, respectively. All assays were recorded with video cameras (25 frames per second, resolution: 1280 × 1024) (acA 1300-60gm GigE, Basler, Ahrensburg, Germany; lens: C-mount 4–8 mm, Computar, Tokyo, Japan) placed on each side of the water bath, and water temperature was monitored every minute using a data logger (iButton, Maxim Integrated, San Jose, CA, USA). To avoid compromising the video quality, we did not use stimuli to provoke movement of test animals during cold or heat exposure.

#### 2.3.3. Manual Assessment of Video Recordings

**Figure 1:**
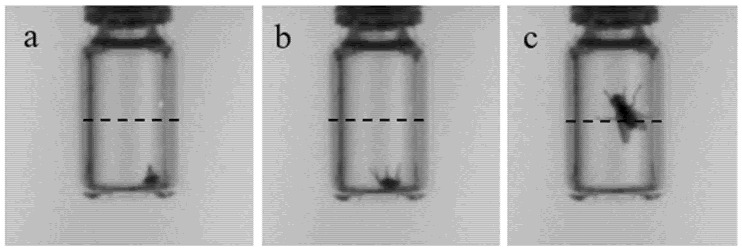
Illustration of the traits used to describe thermal tolerances of flies. The photos originate from an analysed video recording and show a housefly (*M. domestica*) progressively recovering from chill coma. Initially, the fly is still in coma (**a**). Hereafter, the fly starts moving its legs (termed “first movement”) (**b**) and finally crosses the middle of the vial by vertical movement (termed “first middle cross”) (**c**). Likewise, in the static high-temperature and dynamic assays, last middle cross and last movement were assessed. The traits were assessed in houseflies and black soldier flies (*H. illucens*) using both a manual and an automated phenotyping method.

CCRT, HKDT and CTmax were measured by visual observations of two traits: (i) the time point of the first (CCRT) or last (HKDT and CTmax) movement of an appendage (leg/wing/antennae etc.), termed first or last movement, and (ii) the time point of the first (CCRT) or last (HKDT, CTmax) time a fly crossed the middle of the vial with any part of the body, termed first or last middle cross (Figure 1). Observations of first and last movements indicated when a fly recovered from chill coma or entered heat coma/death, respectively. First and last middle cross was a measure representing the first or last time point or temperature at which flying, or vertical walking, was not impaired by thermal stress. The described traits were scored by a single observer visually inspecting the video recordings. The observer was allowed to reassess videos several times, and to pause and change the video speed if needed. For each vial, the centre line was identified with a ruler. If flies did not move or cross the middle of the vial, the fly was excluded from the analysis (Table S2).

#### 2.3.4. Automated Assessment of Video Recordings

CCRT, HKDT and CTmax were further estimated from the recordings using EthoVision XT for video tracking. The automated assessment of video recordings allowed us to measure the following traits; first (CCRT) or last (HKDT and CTmax) movement, first (CCRT) or last (HKDT and CTmax) middle cross and the distance moved throughout the assay for each fly. First/last movements were determined as the first/last non-zero values of activity. Similarly, first/last middle crosses were determined as the first/last times flies were detected in the upper part of the vial. Distance moved, activity and position in the vial were automatically recorded by EthoVision. For each stress tolerance assay, tracking data obtained from the 30 flies positioned on each side of the water bath were merged into one dataset, and recorded traits were summed at intervals of 15 s. The distance moved by each fly was registered as the distance travelled for the body centre of each fly between frames. Data were expressed in centimetres (a calibration scale was added to the software). For each species, assay and treatment group, the distance moved was assessed both by time profiles with data from the 15 s intervals and by summing data for the entire duration of the assay. The video recordings varied in duration between the assays, wherefore summarised distance moved is not comparable across assays and species. Species-specific background noise, originating from changes in lighting, bubble formation, minor disturbance of the cameras etc., was quantified using video recordings of the static high-temperature assays in a time window where all real activity had clearly ceased (Figures S4–S7). The maximum value of distance moved and activity in this time window was identified and subtracted from all data for every stress response assay, and sub-zero values were corrected to zero. This filtering step was performed in R v.4.0.3 [27]. If a fly did not display a trait (for instance, a fly that did not cross the middle of the vial), the fly was excluded from the analysis (Table S2). In the static high-temperature assay with the black soldier fly, a technical error on the video material resulted in data being excluded after a video duration of 1500 s, and therefore last movement could not be evaluated using automated assessment in this assay. Since all flies exhibited last middle cross before the 1500 s (evaluated from the video recordings), this trait was still included in the analysis. In the dynamic assays, the temperature at a given time point for last movement and last middle cross was determined using linear regression models fitted to measurements of the water temperature over the duration of the assays (Figure S8).

### 2.4. Statistical Analysis

All statistical analyses were performed in R v.4.0.3 [27]. The relationship between the manually and automatically obtained estimates of larval body size was investigated using correlation analysis. The assumptions of parametric testing (Pearson correlation), including normality, absence of extreme outliers, homoscedasticity and linearity, were evaluated for size estimates of the black soldier fly and housefly larvae. Normality was evaluated using QQ-plots and extreme outliers were identified using the interquartile range criterion (>Q_3_ + 1.5 × IQR or <Q_1_ − 1.5 × IQR). Homoscedasticity and linearity were evaluated by visual inspection of the scatterplots with body mass on the *x*-axis and surface area on the *y*-axis. These assumptions were met for the black soldier fly, wherefore the relationship between body mass and surface area was analysed using Pearson (raw data) correlation. As housefly larval size estimates did not meet the assumptions of parametric testing, the relationship between body mass and surface area was analysed using Spearman (rank-ordered) correlation. Goodness of fit was evaluated using the Spearman correlation coefficient (r_s_) or the Pearson correlation coefficient (r and R^2^). The variation in size estimates of technical replicates (repeated weighing or estimation of surface area of the same individual, *n* = 3) was quantified using the coefficient of variation, CV (%). Normality and outliers for CV data were assessed using QQ-plots and the interquartile range criterion. Since data did not meet the assumptions for parametric testing, differences in CV between manual and automated size estimates were investigated using the Wilcoxon signed rank test.

Data on first/last movement, first/last middle cross and distance moved were log-transformed (ln (x + 1) to handle zero-values). For the traits first/last movement and first/last middle cross, a two-way ANOVA with an interaction term was applied to analyse the effects of the categorical variables treatment (acclimation) and method (manual or automated phenotyping). Differences in the distance moved between flies exposed to different acclimation treatments were assessed using one-way ANOVA. ANOVA residuals were normally distributed, as evaluated from visual inspections of residual plots. For all comparisons, a significance level of 0.05 was used. Standard errors of measured traits from each stress tolerance assay were calculated using the “se” function in the R-package “goeveg” [28]. All plots presented in this study were produced using the R-package “ggplot2” [29].

## 3. Results

### 3.1. Contrasting Manual and Automated Phenotyping Methods

#### 3.1.1. Larval Body Size

**Figure 2:**
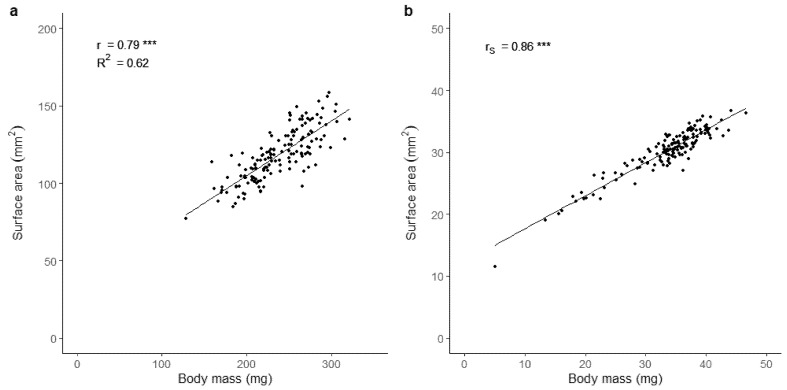
Correlations between body mass and surface area in larvae of the black soldier fly (*H. illucens*) (**a**) and the housefly (*M. domestica*) (**b**). Goodness of fit is indicated by the Pearson correlation coefficients r and R^2^ (**a**) and the Spearman correlation coefficient r_s_ (**b**). Significance level is illustrated as *** (*p* < 0.001). *n* = 150 larvae for each species.

There was a highly significant positive correlation between automated (surface area, mm^2^) and manual (body mass, mg) larval body size estimations for both the black soldier fly (r = 0.79; *p* < 0.001) and the housefly (r_S_ = 0.86; *p* < 0.001) (Figure 2). Furthermore, we observed a significant difference between the coefficient of variation in technical replicates obtained with the automated and the manual phenotyping methods in both species (*p* < 0.001); the largest variation was obtained with the automated method (black soldier fly: mean CV_automated_ = 6.45%, mean CV_manual_ = 0.21%; housefly: mean CV_automated_ = 1.83%, mean CV_manual_ = 1.04%). The time spent on three rounds of manual size estimation for 150 larvae was approximately 22 and 28 min, while the automated surface area estimation required 4 and 5 min for the black soldier fly and the housefly, respectively.

#### 3.1.2. Thermal Tolerances

##### Black Soldier Fly

**Figure 3:**
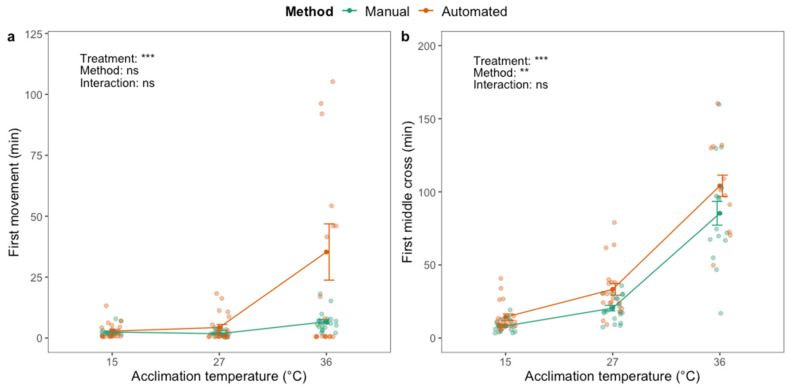
First movement (**a**) and first time crossing the middle of the vial (**b**) after chill coma (2 h at 0 °C) of adult black soldier fly males (*H. illucens*) using manual (green) and automated (orange) phenotyping methods. Dark green and orange dots and brackets indicate means and standard errors. Prior to the assessment of chill coma recovery time, flies were acclimated for 48 h at three different temperatures: 15, 27 and 36 °C. The results from two-way ANOVA with an interaction term are included in the plots (categorical variables are acclimation treatment and phenotyping method). Data were log transformed prior to applying the ANOVA. The significance levels are illustrated as ns (not significant), ** (*p* < 0.01) and *** (*p* < 0.001).

For the black soldier fly, there was a significant effect of thermal acclimation on first movement (F_2,119_ = 11.77; *p* < 0.001) and first middle cross (F_2,104_ = 156.90; *p* < 0.001) after chill coma (CCRT). First movement and first middle cross occurred later in flies acclimated at high temperature (Figure 3). There was no effect of phenotyping method on first movement (Figure 3a). First middle cross was, on average, detected >1 h later for flies acclimated at the highest temperature compared to the other treatment groups (Figure 3b), and five or nine of these flies (out of 20) never crossed the middle of the vial, depending on the phenotyping method (Table S2). There was a significant effect of phenotyping method on first middle cross (F_1,104_ = 10.55; *p* < 0.01), which was detected earlier when using the manual method compared to the automated method (Figure 3b). There was no interaction effect between acclimation treatment and phenotyping method.

Acclimation treatment also significantly affected last middle cross (F_2,118_ = 11.93; *p* < 0.001) and last movement (F_2,59_ = 41.2; *p* < 0.001) when assessing the HKDT, and both traits were first detected in flies acclimated at low temperature (Figure S9). For all three acclimation groups, last movement was detected at later time points when assessed manually compared to automatically (Figure S9a), and the effect of phenotyping method on last middle cross was significant (F_1,118_ = 12.09; *p* < 0.001). There was a significant interaction effect between treatment and method for last middle cross (F_2,118_ = 6.22; *p* < 0.01).

**Figure 4:**
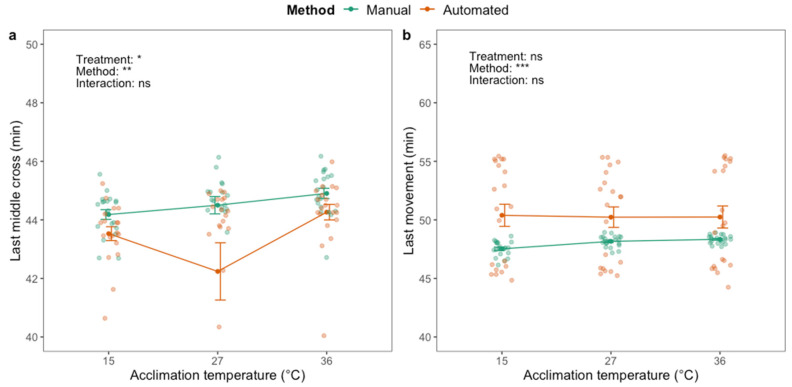
The temperature where adult black soldier fly males (*H. illucens*) last (**a**) cross the middle of the vial and (**b**) move, when the temperature is ramped from 27 °C using manual (green) and automated (orange) phenotyping methods. Dark green and orange dots and brackets indicate means and standard errors. Prior to the assessment of critical thermal maximum, flies were acclimated for 48 h at three different temperatures: 15, 27 and 36 °C. The results from two-way ANOVA with an interaction term are included in the plots (categorical variables are acclimation treatment and phenotyping method). Data were log transformed prior to applying the ANOVA. The significance levels are illustrated as ns (not significant), * (*p* < 0.05), ** (*p* < 0.01) and *** (*p* < 0.001).

When assessing the CTmax, there was a significant effect of acclimation treatment on last middle cross (F_2,116_ = 3.56; *p* < 0.05) (Figure 4a) but not on last movement (Figure 4b). Heat-acclimated flies exhibited last middle cross at a higher temperature than flies from the other acclimation groups. There was a significant effect of the method on both last middle cross (F_1,116_ = 9.16; *p* < 0.01) and last movement (F_1,119_ = 16.34; *p* < 0.001), where last middle cross was observed at a lower temperature, and last movement at a higher temperature, with the automated phenotyping method compared to the manual method (Figure 4).

##### Housefly

For houseflies, there was a significant effect of acclimation treatment on first movement (F_2,119_ = 5.18; *p* < 0.01) and first middle cross (F_2,117_ = 5.68; *p* < 0.01) after chill coma (CCRT), where flies acclimated at low temperature exhibited both traits later compared to flies acclimated at higher temperatures (Figure S10). There was no effect of phenotyping method on first movement or first middle cross.

**Figure 5:**
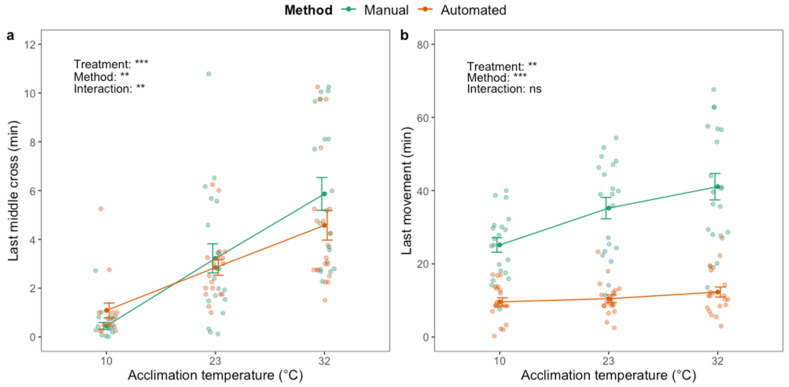
Last time crossing the middle of the vial (**a**) and last movement (**b**) of adult housefly males (*M. domestica*) after exposure to 45 °C using manual (green) and automated (orange) phenotyping methods. Dark green and orange dots and brackets indicate means and standard errors. Prior to the assessment of heat knockdown time, flies were acclimated for 48 h at three different temperatures: 10, 23 and 32 °C. The results from two-way ANOVA with an interaction term are included in the plots (categorical variables are acclimation treatment and phenotyping method. Data were log transformed prior to applying the ANOVA. The significance levels are illustrated as ns (not significant), ** (*p* < 0.01) and *** (*p* < 0.001).

A significant effect of acclimation treatment on last middle cross (F_2,111_ = 67.44; *p* < 0.001) and last movement (F_2,119_ = 5.75; *p* < 0.01) was identified when assessing the HKDT; flies acclimated at high temperature exhibited both traits at later time points than flies in the other acclimation groups (Figure 5). There was a significant effect of the method on last middle cross (F_1,111_ = 9.24; *p* < 0.01) and an interaction effect between acclimation treatment and method (F_2.111_ = 7.00; *p* < 0.01). Last middle cross of the cold-acclimated flies was observed later with the automated compared to the manual phenotyping method (Figure 5a), and six (out of 20) of the cold-acclimated flies never crossed the middle of the vial (Table S2). The opposite was observed for the heat-acclimated flies as they exhibited last middle cross at later time points than flies from the other treatment groups. Last movement was significantly affected by phenotyping method (F_1,119_ = 128.40; *p* < 0.001) and was observed up to ~30 min later when assessed manually compared to automatically (Figure 5b). There was no interaction effect between acclimation treatment and phenotyping method on last movement.

When assessing the CTmax, acclimation treatment significantly affected the temperature of the last middle cross (F_2,115_ = 4.08; *p* < 0.05). The last middle cross was observed at a lower temperature in flies acclimated at an intermediate temperature, compared to flies acclimated at lower and higher temperatures, which exhibited last middle cross at approximately the same temperature (Figure S11). There was no effect of phenotyping method on last middle cross. Acclimation treatment did not influence the temperature at which last movement occurred, but there was a significant effect of phenotyping method for this trait (F_1,117_ = 32.80, *p* < 0.001), with the automated method yielding higher temperature tolerances for all treatment groups (Figure S11b).

### 3.2. Automated Asssessment of Thermal Stress Responses

#### 3.2.1. Black Soldier Fly

**Figure 6:**
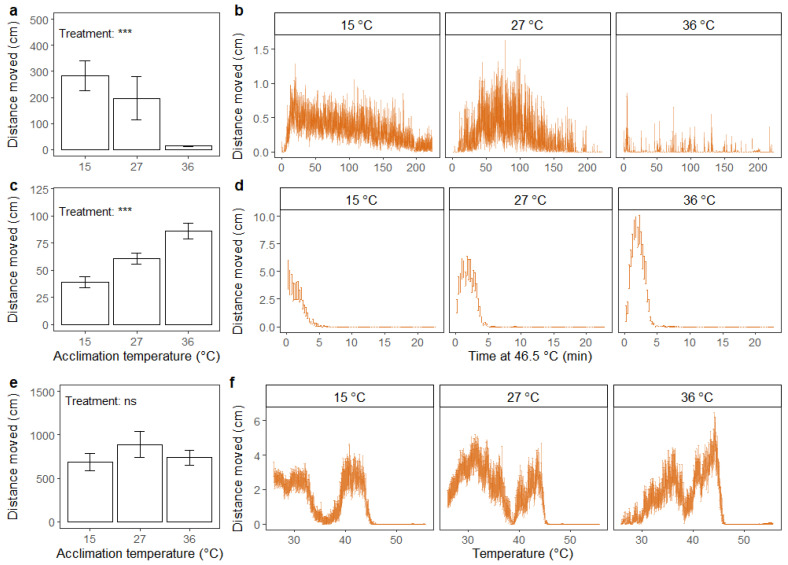
Distance moved (cm) summed across the duration of the assay (**a**,**c**,**e**) (mean ± standard error) and accumulated every 15 s (**b**,**d**,**f**) (mean ± standard error) for adult black soldier fly males (*H. illucens*). Assays include recovery after 2 h of chill coma (0 °C) (**a**,**b**), exposure to static high temperature (46.5 °C) (**c**,**d**) and exposure to thermal ramping from 27 °C (0.2 °C min^−1^) (**e**,**f**). Prior to running the assays, flies were acclimated for 48 h at three different temperatures: 15, 27 and 36 °C. The results from one-way ANOVA on the effect of acclimation treatment on summed distance moved are included in the bar plots with significance levels illustrated as ns (not significant) and *** (*p* < 0.001). Data were log transformed prior to applying the ANOVA.

For black soldier flies, the effect of acclimation treatment on total distance moved after chill coma was significant (F_2,59_ = 26.72; *p* < 0.001), where heat-acclimated flies moved less (Figure 6a,b). In addition, the highest level of fly activity (peak in distance moved, Figure 6b) after chill coma seemingly occurred at an earlier time point for flies acclimated at low temperature compared to flies acclimated at an intermediate temperature. Flies acclimated at high temperature had stable low levels of movement.

For flies exposed to static high temperature, the effect of acclimation treatment on the distance moved was significant (F_2,59_ = 12.93; *p* < 0.001), and cold-acclimated flies moved less under heat stress compared to heat-acclimated flies (Figure 6c). For cold-acclimated flies, the distance moved apparently peaked at the start of the assay and decreased hereafter (Figure 6d). For flies acclimated at intermediate and high temperatures, there was an initial increase in distance moved, and the peak of movement was higher for heat-acclimated flies (Figure 6d).

In the dynamic assay, there was no effect of acclimation treatment on total distance moved (Figure 6e). However, when visually inspecting movements throughout the duration of the assay, differences in peaks in the distance moved between treatment groups were evident (Figure 6f). For the heat-acclimated flies, the peaks seemingly occurred at a higher temperature than for flies in the other treatment groups. From Figure 6f, it is also apparent that flies entered heat coma at a lower temperature than the 50 °C estimated in previous analyses (Figure 4b).

#### 3.2.2. Housefly

**Figure 7:**
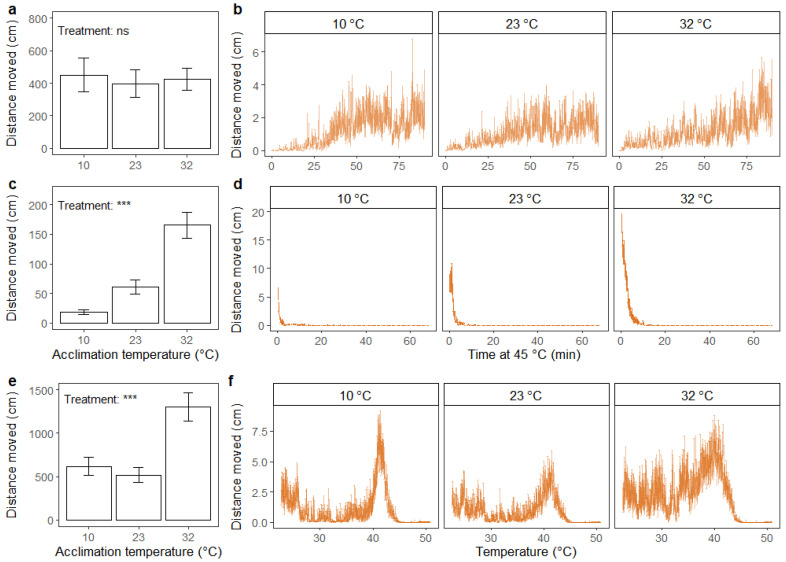
Distance moved (cm) summed across the duration of the assay (**a**,**c**,**e**) (mean ± standard error) and accumulated every 15 s (**b**,**d**,**f**) (mean ± standard error) for adult housefly males (*M. domestica*). Assays include recovery after 2 h of chill coma (0 °C) (**a**,**b**), exposure to static high temperature (45 °C) (**c**,**d**) and exposure to thermal ramping from 23 °C (0.2 °C min^−1^) (**e**,**f**). Prior to running the assays, flies were acclimated for 48 h at three different temperatures: 10, 23 and 32 °C. The results from one-way ANOVA on the effect of acclimation treatment on summed distance moved are included in the bar plots with significance levels illustrated as ns (not significant) and *** (*p* < 0.001). Data were log transformed prior to applying the ANOVA.

For the housefly, there was no effect of acclimation treatment on the total distance moved after chill coma (Figure 7a). Flies from the three treatment groups showed similar patterns of movement throughout the duration of the assay, where the distance moved steadily increased throughout the experiment (Figure 7b).

Significant effects of acclimation treatment on total distance moved were found in the static high-temperature assay (F_2,59_ = 32.43; *p* < 0.001), where flies acclimated to a high temperature moved greater distances than flies from the intermediate temperature acclimation treatment group, and vice versa for the flies acclimated to the lowest temperature (Figure 7c). Throughout the assay, the cold-acclimated flies ceased to move earlier than flies from the other treatment groups (Figure 7d).

In the dynamic assay, there was a significant effect of acclimation treatment on total distance moved (F_2,58_ = 9.81; *p* < 0.001), where flies acclimated at high temperature showed higher total distance moved than flies from the other acclimation treatments (Figure 7e). Flies acclimated at low and intermediate temperatures exhibited relatively similar patterns of movement throughout the duration of the assay, with a small peak in the beginning, followed by a decrease and finally a peak of maximum movement before heat coma (Figure 7f). This pattern was not as obvious for the heat-acclimated flies, whose level of movement seemed to increase steadily over the entire test period until a peak was reached before heat coma (Figure 7f).

## 4. Discussion

### 4.1. Estimates of Larval Size

Larval size is often determined by weighing larvae individually or by deriving body mass estimates from group measurements. In this latter situation, group measures do not capture the individual mass heterogeneity of the given sample and may represent an inaccurate estimate of individual body size. Even though more information can be obtained from individual weighing, it is a tedious and time-consuming process. Here, we demonstrate a method allowing high-throughput phenotyping of individual larval size using a simple setup relying on an image analysis software for size estimation. EthoVision XT was used in our study but similar results should be obtainable using alternative tracking software options (see Robie et al. [30] for suggestions on free open- and closed-source tracking software packages).

The strong correlation between larval surface area and body mass found in our study suggests that the automated phenotyping method is a valid and reliable alternative to manual weighing. Importantly, this method allowed for assessment of the body size of five times more larvae within the same amount of time compared to manual weighing. The use of backlighting ensured a high contrast between the background and the larvae regardless of their colour, underlining that this setup can be applied to a range of morphologically different life stages and species. However, a larger variation in size between replicate measurements of black soldier fly larvae was found with the automated compared to the manual set up, possibly attributable to the flat body of the larvae making the surface area estimate more sensitive to body positioning. Therefore, it is necessary to test the compliance between weight and surface area before applying the automated phenotyping method in experimental work on new species.

Automation of size measurements in small arthropods has previously been demonstrated for collembolans and aquatic invertebrates [4,5,8]. These studies reported strong relationships (R^2^ ≥ 0.90) between length estimates obtained manually or by using novel methods of varying degrees of automation and throughput capacity. Our study distinguishes itself markedly from the above-mentioned studies by focussing on surface area and body mass rather than body length. The use of surface area as a measure of size is less sensitive to the shape of the animals than length, as it is not required to fit a straight line through the longest orientation of the body. As an illustration, Duckworth et al. [8] demonstrated that automatically obtained estimates of body length are more precise for spherical species than for species with elongated bodies.

The degree of automation that we are proposing here is generally higher than in previous published procedures. For instance, the method proposed by Agatz et al. [5] relied on manual analysis of images (i.e., a researcher needed to manually identify the start and end of a measurement by clicking). In our study, size estimates were obtained in real time on un-anaesthetised test animals, and no additional data filtering was needed after individuals were measured. The only manual work that was required prior to the experiment consisted of (i) removing excess rearing substrate from the larvae (also necessary before weighing individuals) before distributing them into well-plates, (ii) acquiring a background image of an empty well-plate and (iii) changing the detection settings in the software to optimise larval detection. We estimated the size of 30 individuals simultaneously regardless of species, which is considerately higher than the throughput reported in other studies. Bánszegi et al. [4] assessed one individual at a time and Duckworth et al. [8] assessed 6 or 24 individuals at a time, depending on the species they assayed. Based on these findings, we argue that large-scale phenotyping of morphological traits in insects will highly benefit from the application of automated systems with high capacity.

### 4.2. Estimates of Stress Tolerance

To our knowledge, this study is the first to compare manually and automatically obtained estimates of stress tolerance in insects for the same traits and individuals. This gives us the advantage of being able to cross-validate the traits scored with each method directly and evaluate the comparability between the two phenotyping methods. In most cases, we found a significant effect of the phenotyping method on the estimate of first/last movement and first/last middle cross. This finding indicates that the automated phenotyping method was not a direct substitute for the manual assessment. The automated method is highly sensitive to experimental disturbances, in particular the formation of bubbles, flickering lighting and small fragments in the water bath. These factors contributed to an increase in the variation in stress tolerance estimates among individuals. We also note that our definition of first/middle cross differed slightly between phenotyping methods: the first/last cross of any body part was measured when assessed manually, while the automated method relied on the detected centre of the body in the vial. This explains some of the difference in the results obtained using the two methods.

Major differences between results obtained from the manual and automated assessment of the last movement were found when estimating the HKDT for the housefly (Figure 5). The much longer time to knockdown obtained with the manual compared to the automated assessment likely resulted from the delicate movements of limbs or mouthparts of the insect, probably emitting an activity signal below the threshold of background noise. Awde et al. [9] experienced similar challenges when attempting to use automated stress tolerance assessments where estimation of the exact HKDT of fruit flies (*Drosophila melanogaster*) was affected by discrepancies of the video recordings. While this issue may hinder the use of the automated method for the estimation of thermal limits, the overall relationships between flies from different treatment groups were generally preserved. Similar observations were made by MacLean et al. [10], who suggested that despite absolute estimates of thermal tolerances not being equal between automated and manual methods applied in their study, the automated method was still applicable for evaluation of thermal acclimation effects in *D. melanogaster*.

An advantage of the manual assessment is immediate data output, since trait values are directly observed, and no further data trimming is required before analysis. Using an observer to phenotype stress tolerance traits is less sensitive to disturbances than using software, where minor discrepancies can drastically influence the resulting trait scores. In addition, manual assessment has low hardware and software requirements, and is therefore economically attainable for most experiments (although labour is also costly). There are several benefits of using video recordings for stress response assessment, regardless of whether software is applied for subsequent analysis or not. Video material can be analysed retrospectively, made open access, played back, paused, test animals can be assessed individually, and trait estimate reliability can be evaluated. Contrary to this, we see potential disadvantages of using manual assessment of stress tolerance traits, including the tediousness of performing direct observations or assessing videos manually, which entails limitations on data throughput. Furthermore, Castañeda et al. [13] described the inter- and intra-researcher repeatability of estimates of morphological, physiological and behavioural traits as a major concern in various biological disciplines and demonstrate how even highly experienced researchers fail to replicate thermal stress tolerance estimates when manually assessing video recordings. Analysing video recordings using software could be a solution to this problem while simultaneously increasing throughput. Data obtained with software require additional filtering and analysis, which increases the time needed to obtain the phenotypes of interest. Data filtering can, however, easily be standardised using algorithms, as highlighted by ours and other studies [6,9,10]. Fine-tuning of the automated phenotyping method presented in this paper is needed if the goal is to obtain measures of delicate end-point traits, such as the last observable movement. This challenge might be overcome by employing machine learning methods to reduce the influence of background noise, and better distinguish the test animals from various disturbances, as suggested by Bruijning et al. [31].

### 4.3. Applying Software for Stress Response Assessment

Often, insect thermal tolerance is described using critical temperatures where individuals die or enter into/recover from coma [23,32,33], possibly because such metrics are relatively easily obtained through direct observations of first/last movement. Even if these thermal tolerance metrics have been linked to the geographical distribution in *Drosophila* species [12,34], it has been suggested that such simple measures can lack an assessment of biologically relevant performance [33,35]. The performance of mobility and behavioural traits, including walking, flying, and fitness traits such as mating behaviour, may be more ecologically relevant and driven by environmental temperatures, unlike those that drive endpoint traits [36,37]. Furthermore, studies have shown that mobility and behavioural traits show effects of acclimation or hardening treatments not seen when using endpoint metrics in traditional stress tolerance assays [22,38]. Mobility traits might be difficult to observe by manual inspection. Moreover, the application of automated phenotyping methods expands the range of information available from thermal tolerance experiments by quantifying response traits such as distance moved, activity and time spent in certain areas of the test arena. As an example, we found that the last movement in houseflies exposed to a gradually increasing temperature (CTmax assay) did not differ between acclimation groups (Figure S11b), thus indicating that acclimation treatment did not affect the heat tolerance in houseflies. However, when assessing the responses to increasing temperature throughout the entire running time of the assay, it was revealed that the total distance moved was higher in heat-acclimated flies than in cold-acclimated flies (Figure 7e). These results clearly imply that high- and low-temperature acclimation impacted the thermal stress response, which was not reflected in the timing of the last movement, thus providing a deeper understanding of the effects of environmental temperature variation (here different acclimation temperatures) on insects’ performance. Lighton and Turner [14] came to a similar conclusion when assessing CTmax values for two species of ants. They found no difference between the species when compared for loss of muscular coordination under heat stress. However, when employing additional information on aerobic capacity, clear differences in heat stress physiology between the two species were found, highlighting that ecologically important responses are not always reflected in simple metrics (see also DeVries et al. [17]). In our study, other complex response patterns observed exclusively with the automated phenotyping method include the effect of acclimation on the double peak in activity observed for both species during thermal ramping (Figure 6f and Figure 7f), and the complete loss of motility in heat-acclimated black soldier flies following cold exposure (Figure 6a,b). These observations emphasise the value of using a holistic approach to predict stress responses in more complex functions which are potentially of more ecological importance than what can be derived from traditional assays (see also Sinclair et al. [33]).

## 5. Conclusions

The present study demonstrates how novel methods can be applied to automate the phenotyping process in different traits and life stages of black soldier flies and houseflies. We found that conclusions drawn from the automated and manual assessment often aligned, and that the automated phenotyping method provided information that we were not able to obtain with the manual methods. Our first hypothesis was accepted, as larval surface area measured with image analysis software was highly correlated with body mass, whilst application of the automated method increased throughput tremendously. We did not obtain the same absolute values of thermal tolerance when applying the image analysis software, and therefore our second hypothesis was rejected. However, the relationship amongst acclimation treatment groups was preserved, thus implying that the automated method can be used to assess relative effects of thermal acclimation. Finally, our third hypothesis was accepted, as the automated method added valuable insights into insect behaviour up until/after the point of heat or chill coma occurrence that could not be observed with manual assessment. When assessing thermal tolerance with the automated phenotyping method presented here, more time is required for data processing than when using manual methods. Nevertheless, we argue that the benefits of using an automated method exceed this cost, especially since continuous observations during the stress tolerance assays can be avoided and because relevant novel biological information can be obtained from the automatic assays. More research is needed to streamline the phenotyping of delicate endpoint traits, but our findings contribute new opportunities within many biological disciplines where accurate and large-scale phenotyping is required.

## Data Availability

The data presented in this study are openly available from the Dryad Digital Repository at doi:10.5061/dryad.cjsxksn5p.

## Supplementary Materials

The following are available online at https://www.mdpi.com/article/10.3390/insects12050380/s1, Figure S1: Experimental setup used for automated assessment of larval body size, Figure S2: Acquisition duration, Figure S3: Experimental setup used in thermal tolerance assays, Figure S4: Removal of background noise (distance moved) in houseflies, Figure S5: Removal of background noise (activity) in houseflies, Figure S6: Removal of background noise (distance moved) in black soldier flies, Figure S7: Removal of background noise (activity) in black soldier flies, Figure S8: Linear regression models of water temperature measurements in thermal ramping assays, Figure S9: Heat knockdown time of black soldier flies, Figure S10: Chill coma recovery time of houseflies, Figure S11: Critical thermal maximum of houseflies, Table S1: Fly ages, Table S2: Number of flies excluded from analyses, Table S3: Survival at different acclimation temperatures (pilot data).
