# Supplementary material for: Contrasting Manual and Automated Assessment of Thermal Stress Responses and Larval Body Size in Black Soldier Flies and Houseflies
Source: Insects. 2021 Apr 22;12(5):380. doi: 10.3390/insects12050380 (PMC8146041; doi:10.3390/insects12050380)
Supplement: Supplementary file 1 [file insects-12-00380-s001.zip › insects-1187123-supplementary.pdf]

Supplementary material for

Contrasting manual and automated assessment of thermal stress responses and larval body size in black soldier flies and houseflies

Stine Frey Laursen [1,*,†], Laura Skrubbeltrang Hansen [2,*], Simon Bahrndorff [1], Hanne Marie Nielsen [2], Natasja Krog Noer [1], David Renault [3,4], Goutam Sahana [2], Jesper Givskov Sørensen [5] and Torsten Nygaard Kristensen [1,6]

[1] Section of Biology and Environmental Science, Department of Chemistry and Bioscience, Aalborg University, Fredrik Bajers Vej 7H, DK 9220 Aalborg, Denmark

[2] Center for Quantitative Genetics and Genomics, Faculty of Technical sciences, Aarhus University, Blichers Allé 20, DK 8830 Tjele, Denmark

[3] University of Rennes, CNRS, ECOBIO (Ecosystémes, biodiversité, evolution) UMR 6553, Rennes, France

[4] Institut Universitaire de France, 1 Rue Descartes, Paris, France

[5] Section for Genetics, Ecology & Evolution, Department of Biology, Aarhus University, Ny Munkegade 116, DK 8000 Aarhus C, Denmark

[6] Department of Agroecology, Aarhus University, Blichers Allé 20, DK 8830 Tjele, Denmark

* Shared first authorship

† Correspondence: sfl@bio.aau.dk

Table of Contents

Figure S1: Experimental setup for automated assessment of larval body size 3

Figure S2: Acquistition duration 4

Figure S3: Experimental setup used in thermal tolerance assays 5

Figure S4: Removal of background noise (distance moved) in houseflies 6

Figure S5: Removal of background noise (activity) in houseflies 7

Figure S6: Removal of background noise (distance moved) in black soldier flies 8

Figure S7: Removal of background noise (activity) in black soldier flies 9

Figure S8: Linear regression models of water temperature measurements in thermal ramping assays 10

Figure S9: Heat knockdown time of black soldier flies 11

Figure S10: Chill coma recovery time of houseflies 12

Figure S11: Critical thermal maximum of houseflies 13

Table S1: Fly ages 14

Table S2: Number of flies excluded from analyses 15

Table S3: Survival at different acclimation temperatures (pilot data) 16

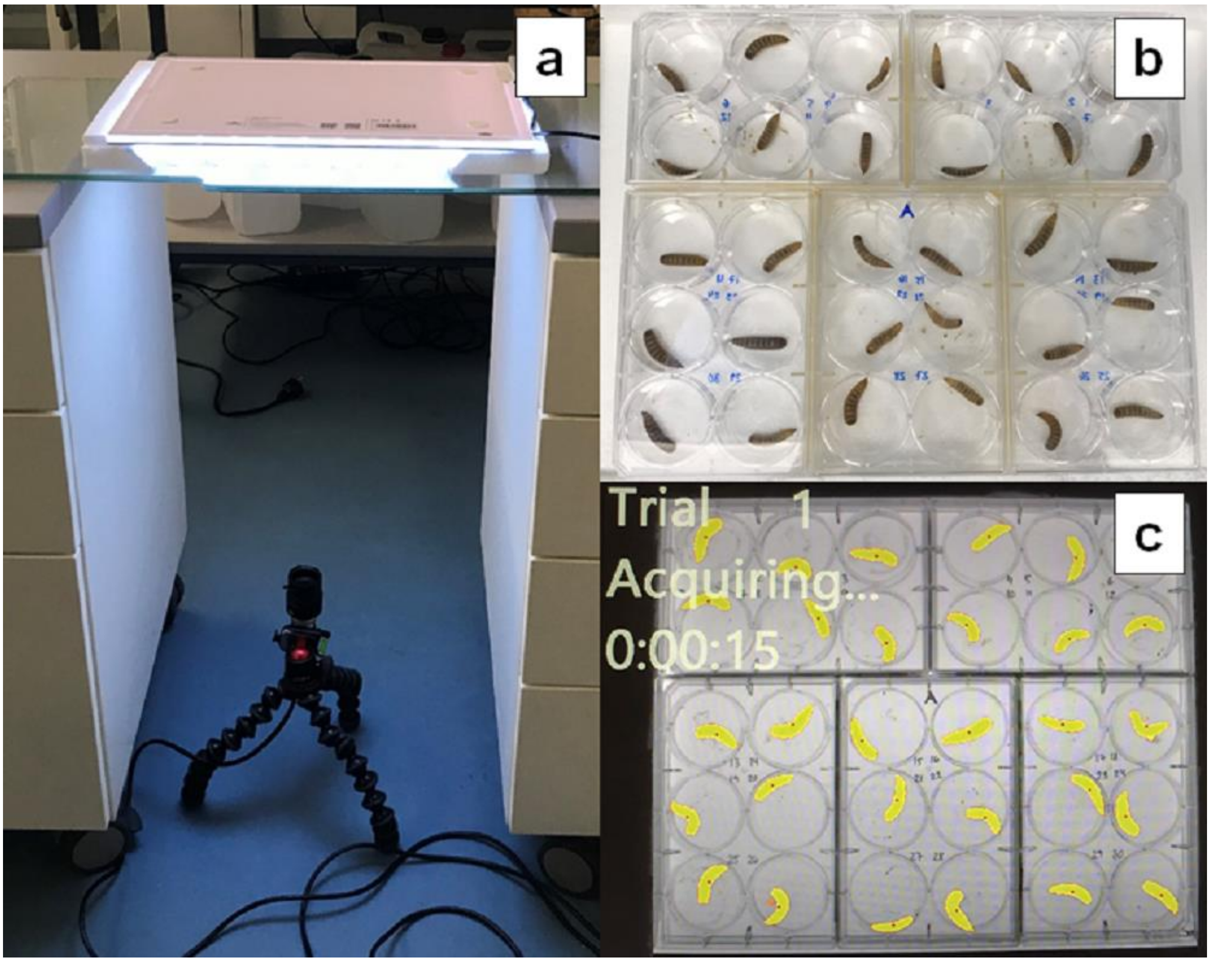

Figure S1 Experimental setup used for automated assessment of larval body size **(a)**. A well-plate containing 30 larvae **(b)** was positioned on a sheet of glass with a light box directly above and a video camera, connected to a computer, underneath. Individual larval body size was estimated using an image analysis software (EthoVision XT) **(c)**.

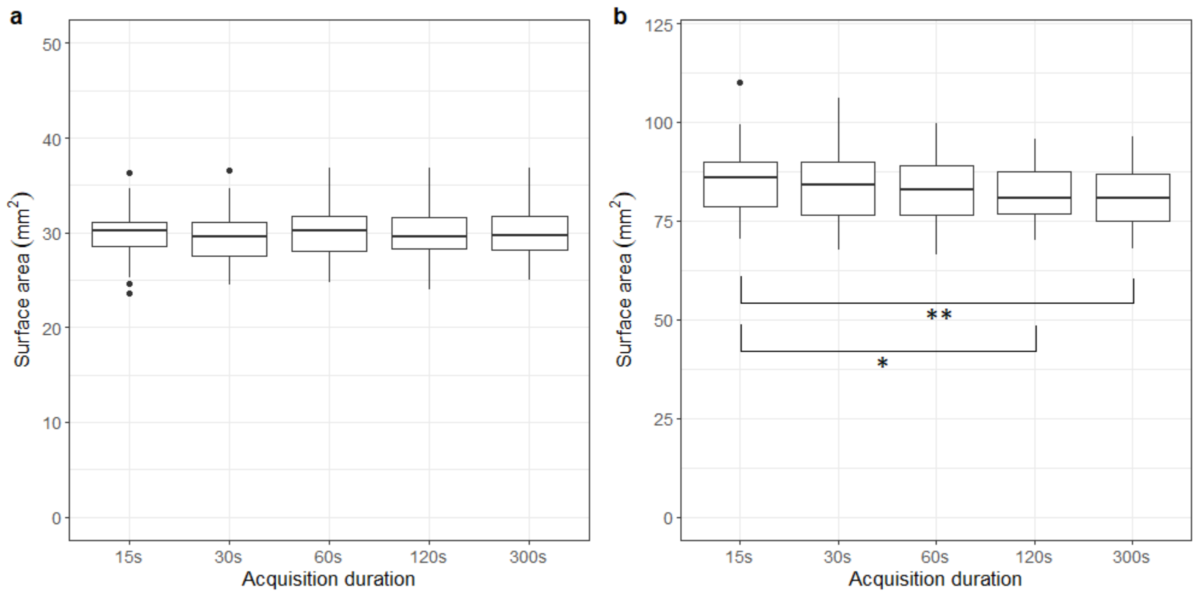

Figure S2 Sizes of housefly (*M. domestica)* **(a)** and black soldier fly (*H. illucens)* **(b)** larvae (n=30) measured with varying acquisition duration (15, 30, 60, 120 and 300 seconds) using an imaging analysis software (EthoVision XT). Sizes were compared using Wilcoxon signed rank test with Bonferroni correction and significance levels are illustrated as * ($p<0.5$) and ** ($p<0.01$). For black soldier flies, larval size obtained over 15 seconds differ from those obtained over 120 and 300 seconds.

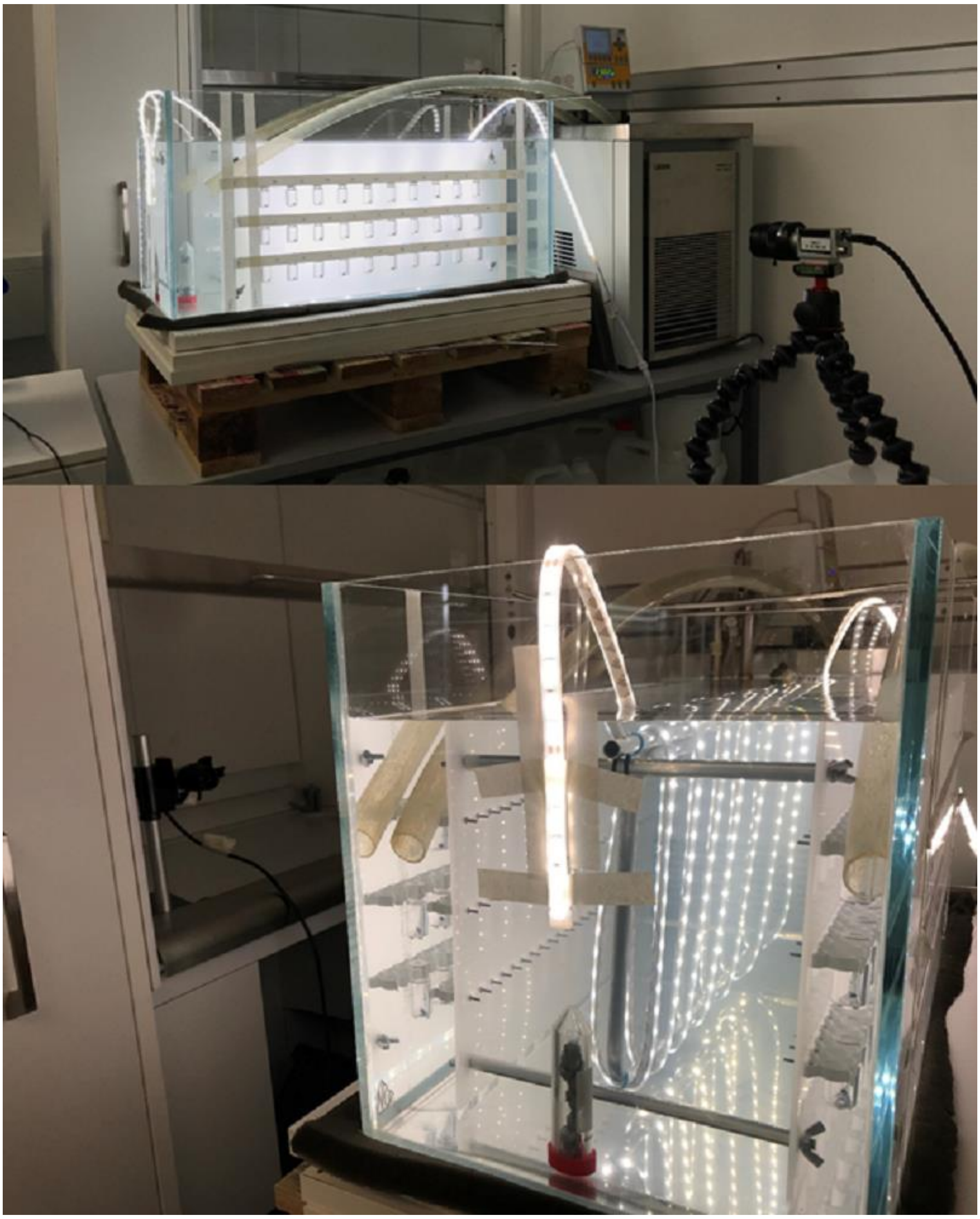

Figure S3 Experimental setup used in all thermal tolerance assays. Flies were separated in screw cap glass vials distributed on a rack constructed of two semi-transparent white acrylic sheets with an LED light source in between. The rack was submerged in a temperature-controlled water bath with a temperature logger monitoring water temperature. All assays were recorded with video cameras placed on each side of the water bath.

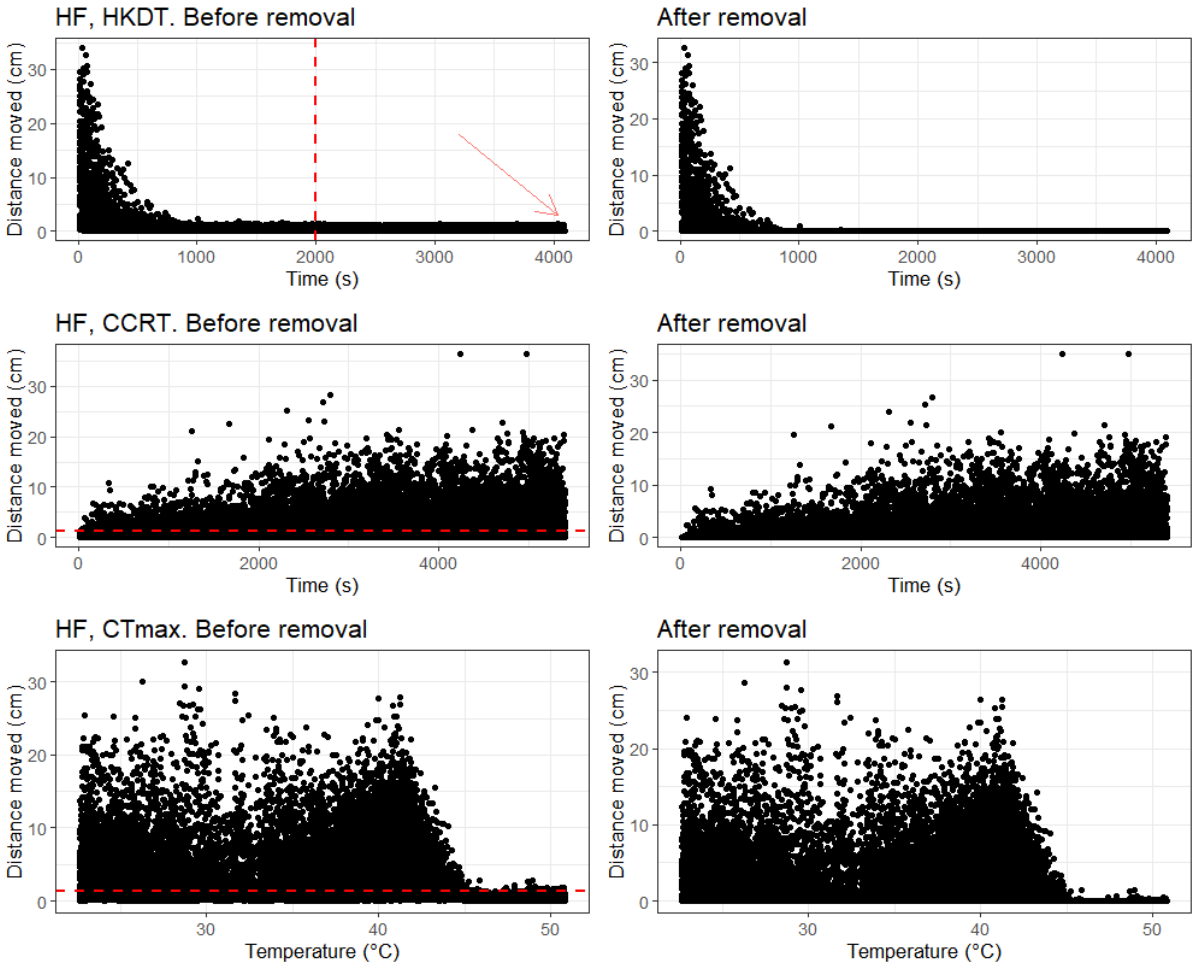

Figure S4 A maximum distance moved-value of 1.38321 cm was determined when real movement had clearly ceased (after 2000 s) in the static high-temperature assay (indicated with a red arrow) of the housefly (HF). This value was subtracted from all distance moved-values in the assay as well as in the remaining assays (indicated with a red dashed line).

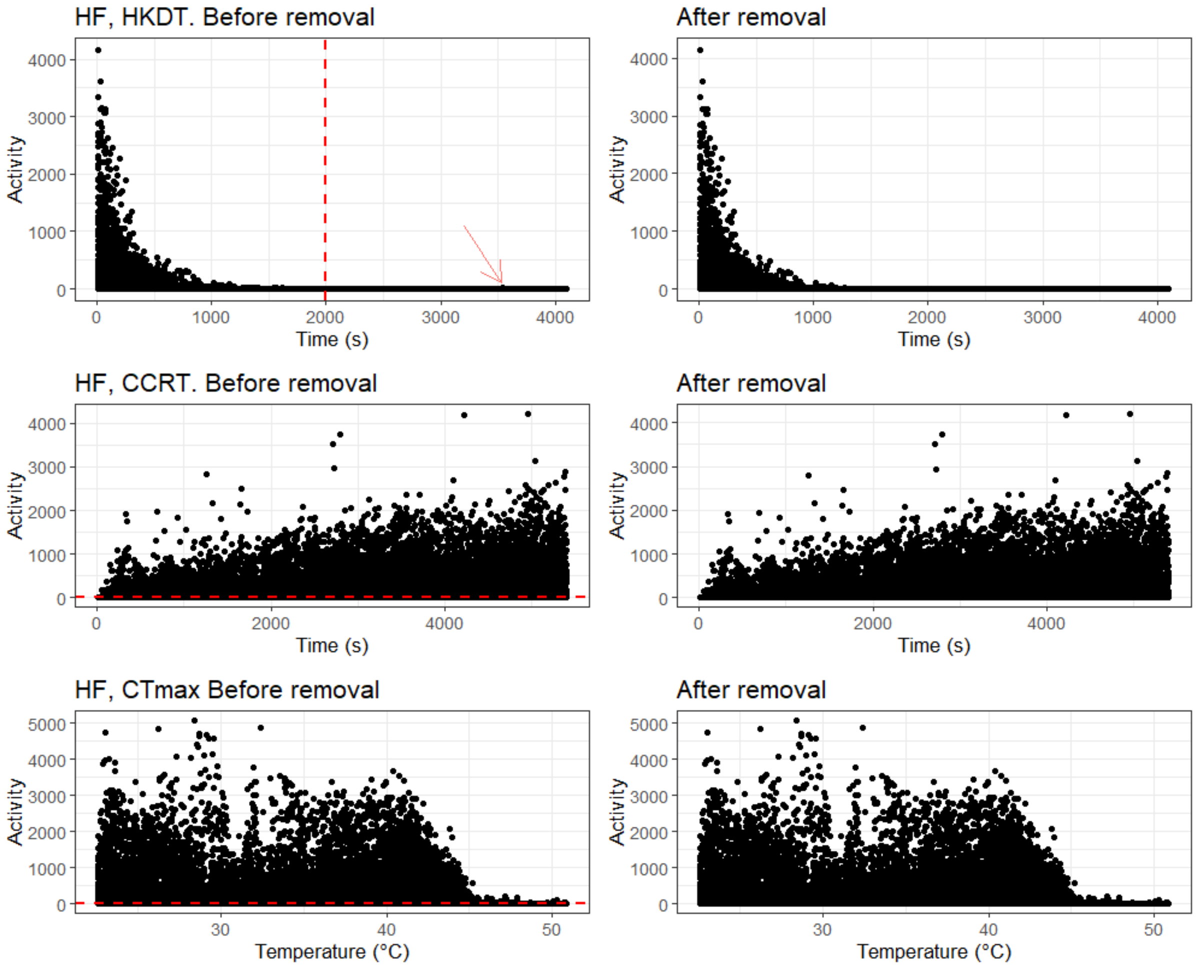

Figure S5 A maximum activity-value of 14.7242 pp was determined when activity had clearly ceased (after 2000 s) in the static high-temperature assay (indicated with a red arrow) of the housefly (HF). This value was subtracted from all activity-values in the assay as well as in the remaining assays (indicated with a red dashed line).

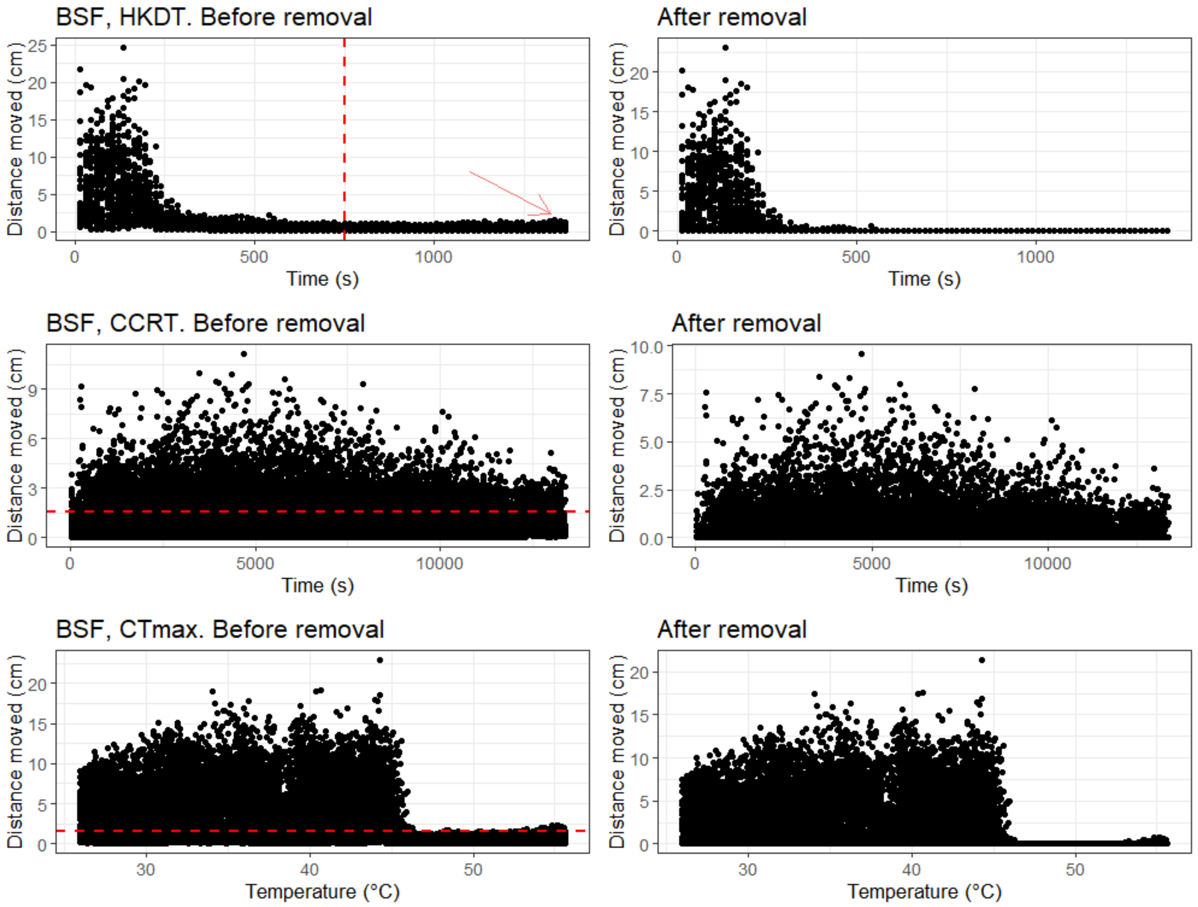

Figure S6 A maximum distance moved-value of 1.54036 cm was determined when real movement had clearly ceased (after 750 s) in the static high-temperature assay (indicated with a red arrow) of the black soldier fly (BSF). This value was subtracted from all distance moved-values in the assay as well as in the remaining assays (indicated with a red dashed line).

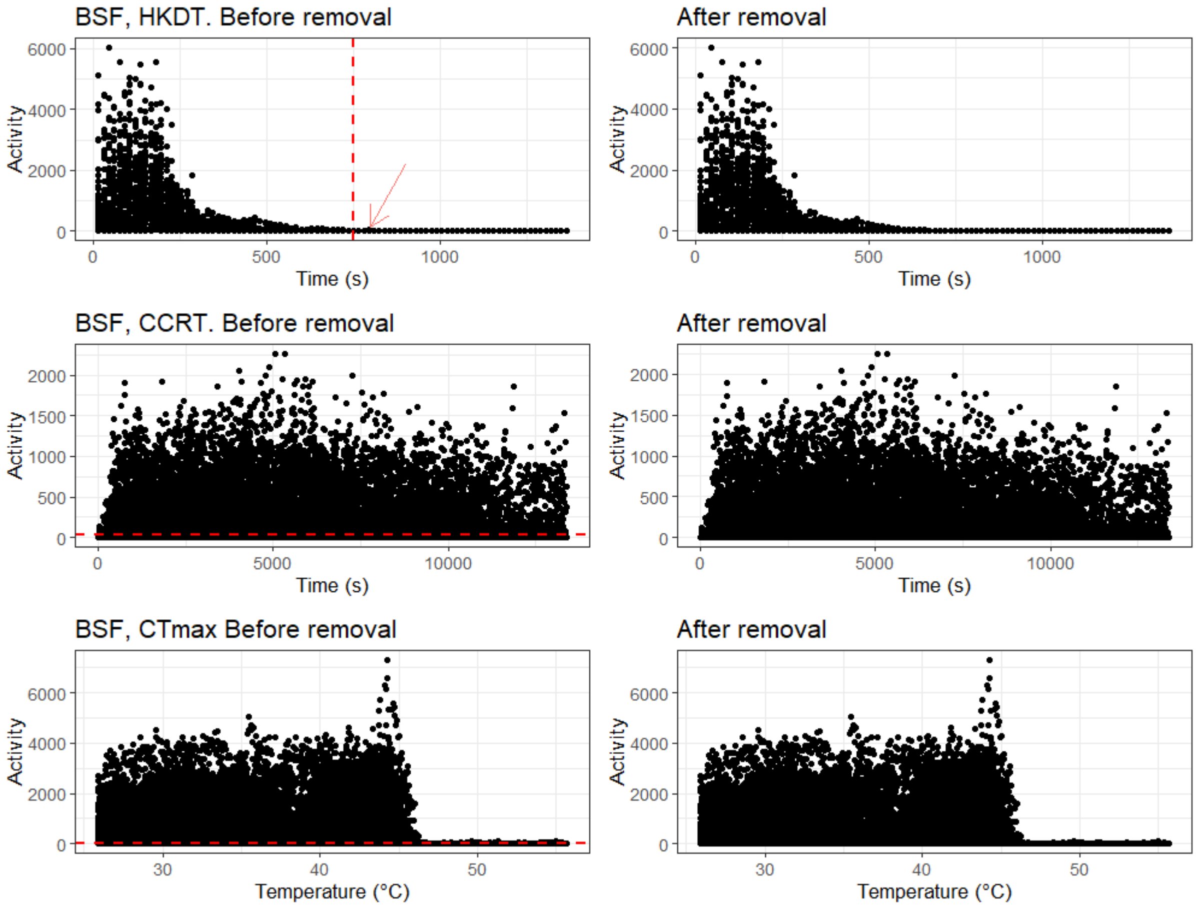

Figure S7 A maximum activity-value of 35.0427 cm was determined when real movement had clearly ceased (after 750 s) in the static high-temperature assay (indicated with a red arrow) of the black soldier fly (BSF). This value was subtracted from all activity-values in the assay as well as in the remaining assays (indicated with a red dashed line).

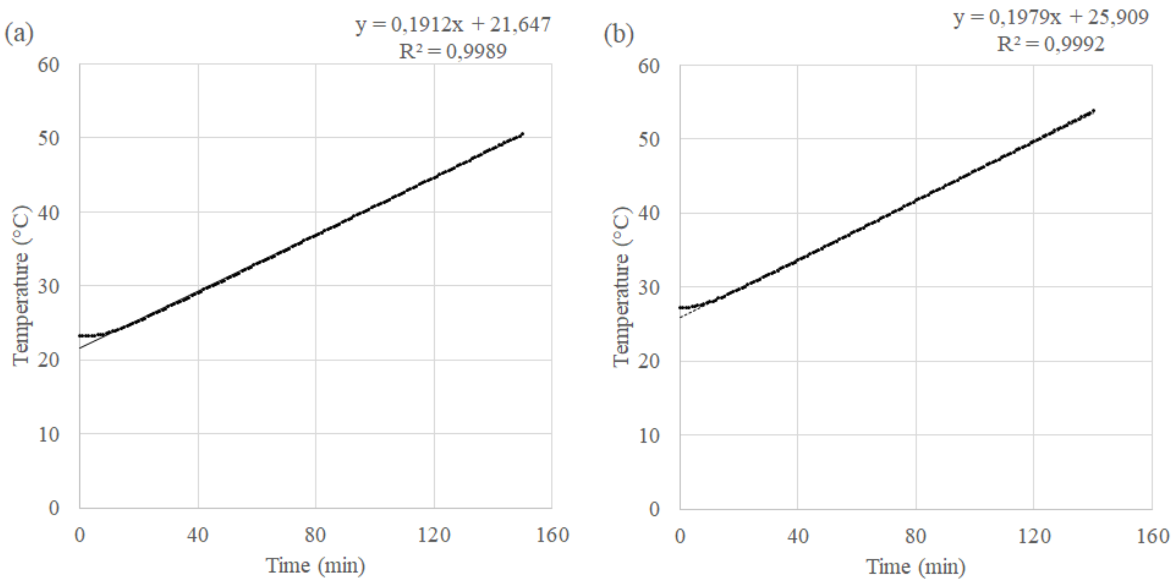

Figure S8 Linear regression models fitted to measurements of the water temperature over the duration of the dynamic (ramping) assays in adult males of the housefly (*M. domestica*) **(a)** and the black soldier fly (*Hermetia illucens*) **(b)**. The linear regression equations were used to identify the temperature at a given time point for last movement and last middle cross of the vial.

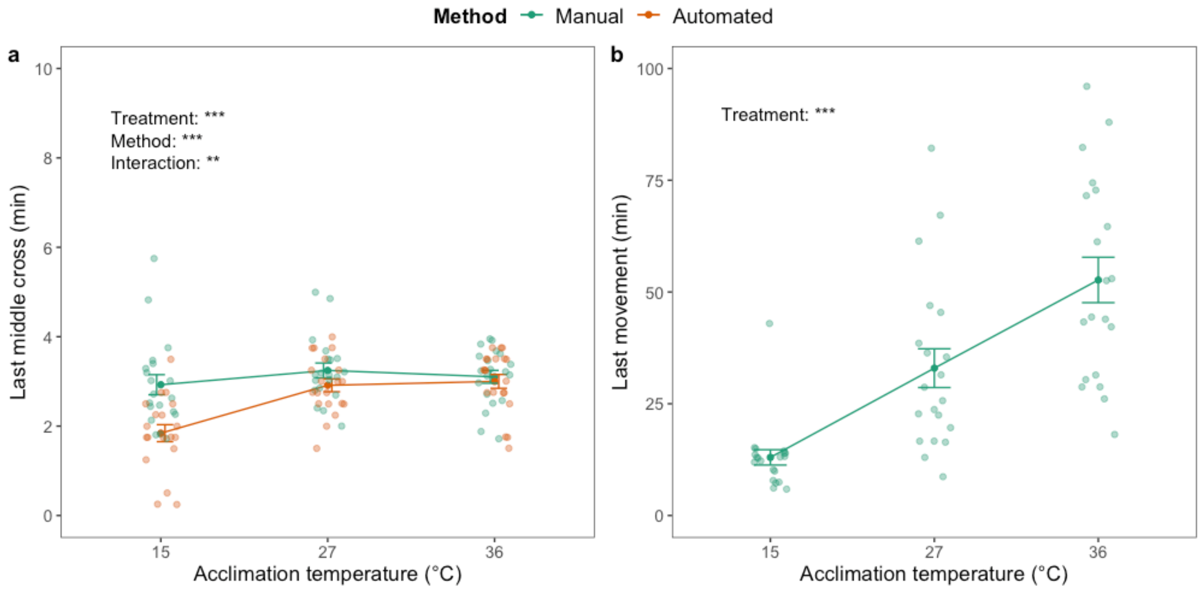

Figure S9 Last time crossing the middle of the vial **(a)** and last movement **(b)** of adult black soldier fly males (*H. illucens)* after exposure to 46.5 ºC using manual (green) and automated (orange) phenotyping methods. Dark green and orange dots and brackets indicate means and standard errors. Prior to the assessment of heat knockdown time, flies were acclimated for 48 hours at three different temperatures: 15, 27 and 36 ºC. Last movement was not assessed automatically due to a technical error on the video recordings. Results from two-way ANOVA with an interaction term are included in **(a)** (categorical variables are acclimation treatment and phenotyping method). Results from one-way ANOVA are included in (b). Data was log transformed prior to applying the ANOVA. The significance levels are illustrated as ** ($p<0.01$) and *** ($p<0.001$).

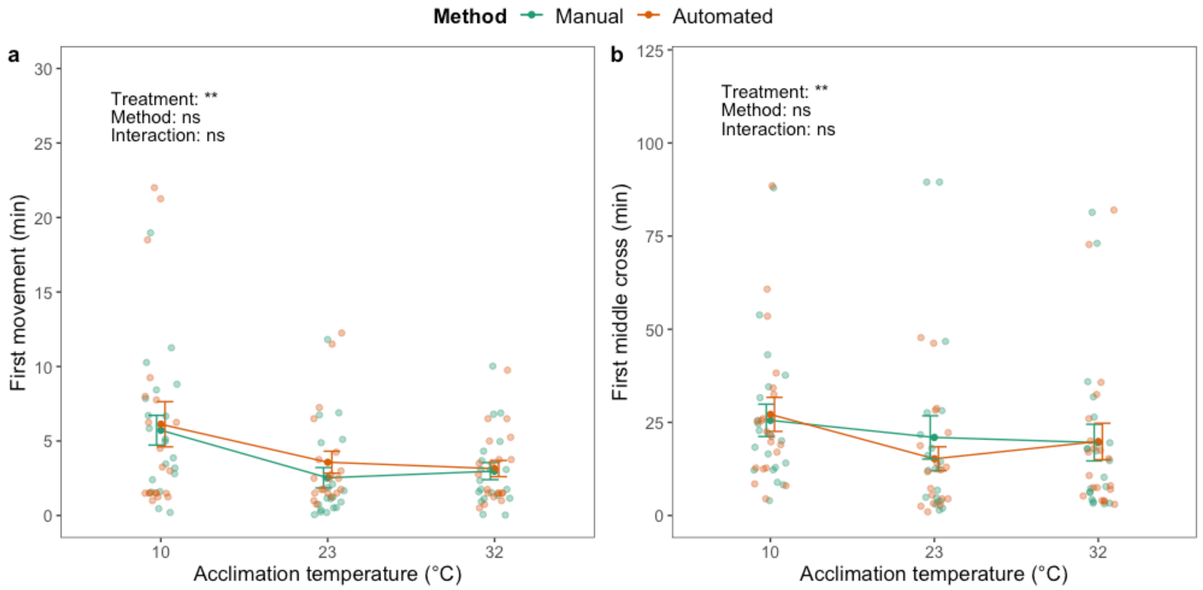

Figure S10 First movement **(a)** and first time crossing the middle of the vial **(b)** after chill coma (2 hours at 0 ºC) of adult housefly males (*M. domestica)* using manual (green) and automated (orange) phenotyping methods. Dark green and orange dots and brackets indicate means and standard errors. Prior to the assessment of chill coma recovery time, flies were acclimated for 48 hours at three different temperatures: 10, 23 and 32 ºC. Results from two-way ANOVA with an interaction term are included in the plots (categorical variables are acclimation treatment and phenotyping method). Data was log transformed prior to applying the ANOVA. The significance levels are illustrated as ns (not significant) and ** ($p<0.01$).

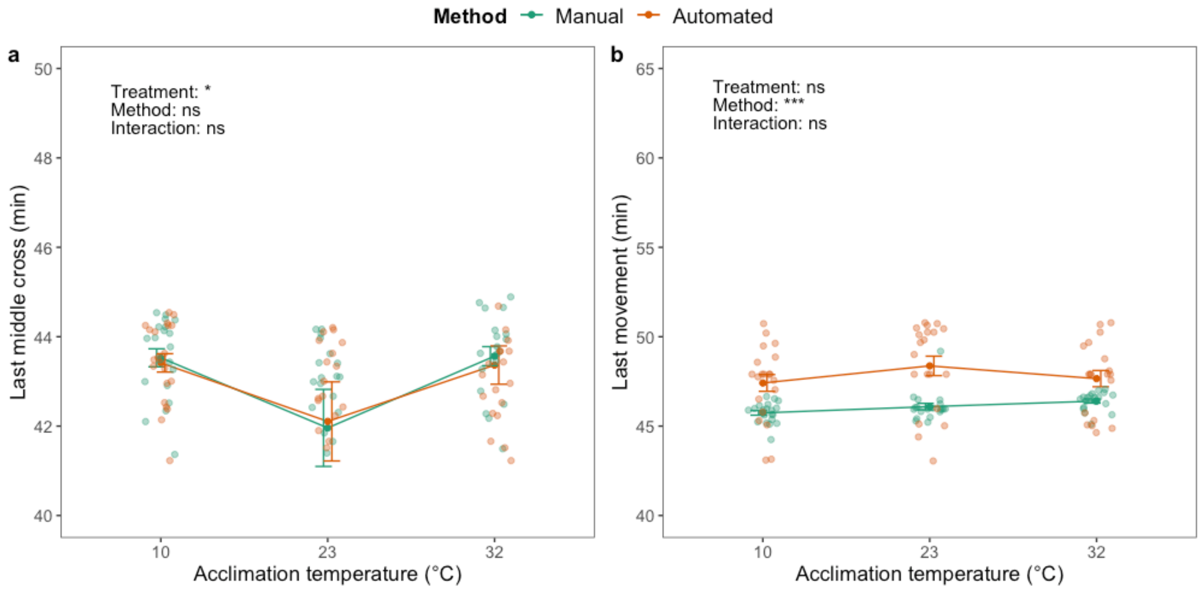

Figure S11 The temperature where adult housefly males (*M. domestica*) last **(a)** cross the middle of the vial and **(b)** move, when the temperature is ramped from 23 ºC using manual (green) and automated (orange) phenotyping methods. Dark green and orange dots and brackets indicate means and standard errors. Prior to the assessment of critical thermal maximum, flies were acclimated for 48 hours at three different temperatures: 10, 23 and 32 ºC. Results from two-way ANOVA with an interaction term are included in the plots (categorical variables are acclimation treatment and phenotyping method). Data was log transformed prior to applying the ANOVA. The significance levels are illustrated as ns (not significant), * (p<0.5) and *** (p<0.001).

Table S1 Age of adult male houseflies *(M. domestica)* and black soldier flies *(H. illucens)* at the start of acclimation and thermal tolerance assays. Cold stress responses were investigated using static assays (chill coma recovery time, CCRT), and heat stress responses were investigated using static (heat knockdown time, HKDT) and dynamic (critical thermal maximum, CTmax) assays.

Assay	Housefly age (hours)		Black soldier fly age (hours)	
	**Acclimation start**	**Assay start**	**Acclimation start**	**Assay start**
**Static low-temperature**	68 ± 6	116 ± 6	13 ± 13	61 ± 13
**Static high-temperature**	84 ± 6	132 ± 6	12 ± 12	60 ± 12
**Dynamic**	80 ± 6	128 ± 6	26.5 ± 26.5	74.5 ± 26.5

Table S2 Number of houseflies (*M. domestica*) and black soldier flies (*H. illucens*) excluded from analyses of first/last movement and first/last middle cross in all assays when assessed manually and automatically. 20 flies were tested in all assays and acclimation temperature combinations. Exclusions of flies from the analyses was most often when a trait was not observed, e.g. 6 houseflies never crossed the middle of the vial in the static high-temperature assay and this was observed when using both the manual and the automated assessment method. In one case, a housefly was not observable during the assay and was thus excluded (dynamic assay, acclimation temperature: 32 ºC). For technical reasons, last movement of black soldier flies was not assessed automatically in the static high-temperature assay.

Species	Assay	Acclimation temperature (ºC)	Manual assessment		Automated assessment	
			First/last movement	First/last middle cross	First/last movement	First/last middle cross
Housefly	Static low-temperature	10	0	0	0	0
		23	0	0	0	0
		32	0	0	0	0
	Static high-temperature	10	0	6	0	6
		23	0	0	0	2
		32	0	0	0	0
	Dynamic	10	0	0	0	0
		23	0	0	0	2
		32	1	1	1	1
Black soldier fly	Static low-temperature	15	0	0	0	0
		27	0	0	0	1
		36	0	5	0	9
	Static high-temperature	15	0	0	-	1
		27	0	0	-	0
		36	0	0	-	0
	Dynamic	15	0	0	0	1
		27	0	2	0	0
		36	0	0	0	0

Table S3 Pilot experiments used to determine acclimation temperatures for black soldier flies (*H. illucens*) and houseflies (*M. domestica*). Adult males (n=10) of the two species were acclimated for 48 hours and the survival was assessed. For black soldier flies, 15 °C and 36 °C were chosen for acclimation treatments as all flies survived. For houseflies, the highest tested temperature (36 °C) resulted in only 40 % survivors, and 32 °C and 10 °C were selected as acclimation temperatures.

	**Temperature (°C)**	**Surviving proportion (n=10)**
**Black soldier fly**	15	1
	19	1
	23	1
	27	1
	30	1
	33	1
	36	1
**Housefly**	10	0,9
	15	1
	20	0,9
	23	0,9
	27	1
	30	1
	32	0,8
	36	0,4
